# miR-27a-3p targeting RXRα promotes colorectal cancer progression by activating Wnt/β-catenin pathway

**DOI:** 10.18632/oncotarget.19635

**Published:** 2017-07-26

**Authors:** Jiangtao Liang, Jianming Tang, Huijuan Shi, Hui Li, Tiantian Zhen, Jing Duan, Lili Kang, Fenfen Zhang, Yu Dong, Anjia Han

**Affiliations:** ^1^ Department of Pathology, The First Affiliated Hospital, Sun Yat-Sen University, Guangzhou, China

**Keywords:** miR-27a-3p, RXRα, Wnt/β-catenin pathway, colorectal cancer

## Abstract

This study aimed to elucidate how miR-27a-3p modulates the Wnt/β-catenin signaling pathway to promote colorectal cancer (CRC) progression. Our results showed that the expression of miR-27a-3p was up-regulated in CRC and closely associated with histological differentiation, clinical stage, distant metastasis and CRC patients’ survival. miR-27a-3p mimic suppressed apoptosis and promoted proliferation, migration, invasion of CRC cells *in vitro* and *in vivo*. Whereas miR-27a-3p inhibitor promoted apoptosis and suppressed proliferation, migration, invasion of CRC cells *in vitro* and *in vivo*. Furthermore, RXRα was the target gene of miR-27a-3p in CRC. miR-27a-3p expression negatively correlated with RXRα expression in CRC tissues. The underlining mechanism study showed that miR-27a-3p/RXRα/Wnt/β-catenin signaling pathway is involved in CRC progression. In conclusion, our findings first demonstrate that miR-27a-3p is a prognostic and/or potential therapeutic biomarker for CRC patients and RXRα as miR-27a-3p targeting gene plays an important role in activation of the Wnt/β-catenin pathway during CRC progression.

## INTRODUCTION

Colorectal cancer (CRC) is one of the most lethal malignancies in which the Wnt/β-catenin pathway plays an important role and is constitutively activated [1]. The retinoid X receptors (RXRs) are nuclear receptors and are members of the superfamily of ligand-inducible transcriptional regulatory factors that mediates the anti-cancer function of retinoids (natural retinoic acids and their synthetic derivatives). RXRs have three isotypes—α, β and γ. It has been reported that retinoic acid receptor (RAR) interacts with β-catenin and inhibits β-catenin-mediated gene transcription [2–8]. We have reported that RXRα directly interacts with β-catenin and regulates β-catenin transcription in CRC cells [9]. Further study shows that suppression of RXRα and aberrant β-catenin expression significantly associates with progression of CRC [10]. Most recently, emerging evidence has suggested that dysregulation of miR-27a-3p may contribute to tumor development and progression in various types of cancer [11–19]. MiR-27a contributes to rhabdomyosarcoma cell proliferation by suppressing RARα and RXRα[20]. Expression of microRNA-27a was increased in CRC stem cells. Knockdown of miR-27a sensitizes CRC stem cells to TRAIL by promoting the formation of Apaf-1-caspase-9 complex [17]. The miR-27a- calreticulin axis affects drug-induced immunogenic cell death in human colorectal cancer cells [18]. However, Bao et al. have reported microRNA-27a is a tumor suppressor in colorectal carcinogenesis and progression by targeting SGPP1 and Smad2 [19]. Our current study is to investigate the role of miR-27a-3p and elucidate how miR-27a-3p modulates the Wnt/β-catenin signaling pathway to promote colorectal cancer (CRC) progression.

## RESULTS

### miR-27a-3p expression correlates with CRC patient survival

**Figure 1:**
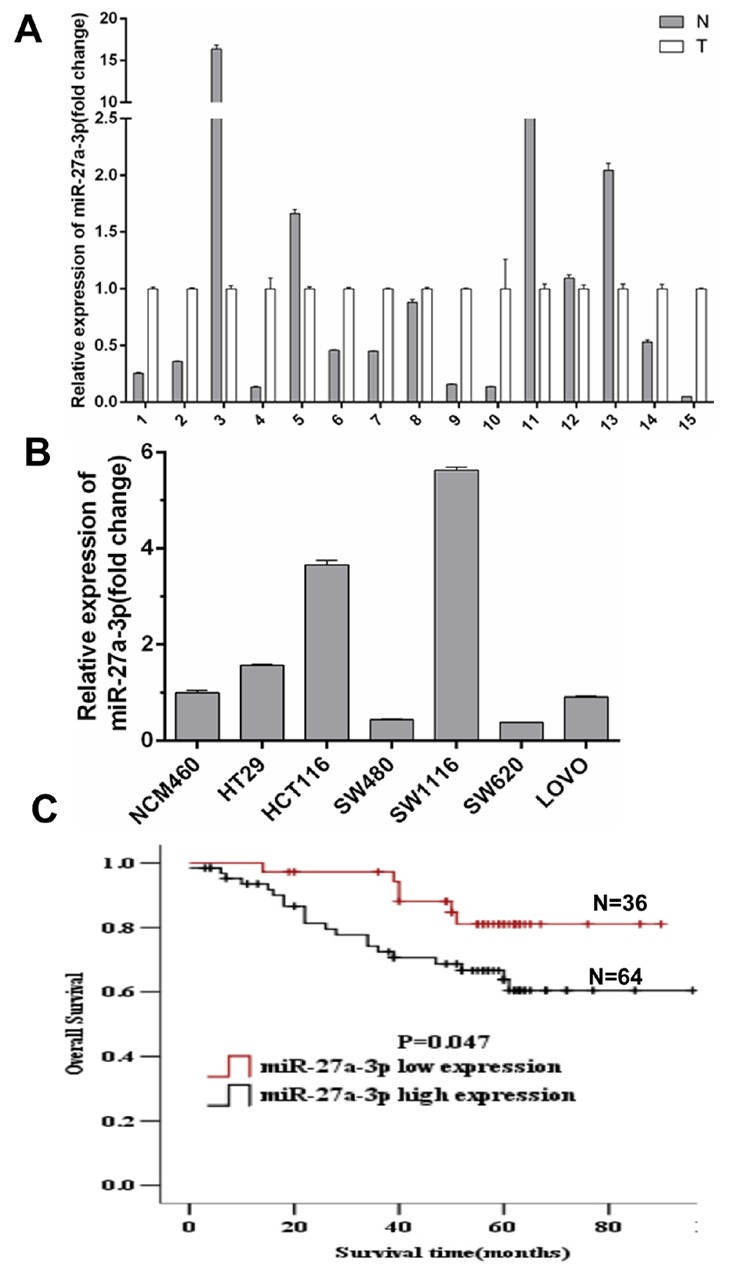
**(A)** miR-27a-3p expression level in 15 paired of fresh CRC samples (T) and adjacent non-tumor colorectal mucosa tissues (N) by quantitative real-time PCR analysis. **(B)** miR-27a-3p expression level in CRC cell lines. Data were normalized against the miR-27a-3p expression level in NCM460 cells. **(C)** Overall survival of CRC patients with different levels of miR-27a-3p expression by Kaplan-Meier analysis.

To determine the significance of miR-27a-3p in CRC, we first performed quantitative real-time PCR on fresh human CRC specimens and their matched adjacent non-tumor colorectal tissues (ANT). miR-27a-3p expression was higher in 10 (67%) of 15 CRC samples compared with their respective ANT (Figure 1A). High miR-27a-3p expression was found in 3 of 6 CRC cell lines including HCT116, HT29, and SW1116 cell lines compared with human colonic epithelial cell line NCM460 (Figure 1B). To further investigate the correlation of miR-27a-3p expression with clinicopathological features of CRC, we detected miR-27a-3p expression in 100 pairs of paraffin-embedded human CRC samples and respective non-tumor colorectal tissues by real-time PCR analysis. The result showed miR-27a-3p expression significantly increased in CRC compared with ANT. Notably, miR-27a-3p expression was associated with the pathological differentiation, Dukes staging, and distance metastasis of CRC (Table 1). The 3-year overall survival rate in CRC patients with miR-27a-3p high expression was 75.3%. However, the 3-year overall survival rate in CRC patients with miR-27a-3p low expression was 87.9%. There was a significant difference (p<0.01). The 5-year overall survival rate (71.1%) was significantly lower in CRC patients with miR-27a-3p high expression than 80.5% of the 5-year overall survival rate in CRC patients with miR-27a-3p low expression (p<0.05). Kaplan–Meier survival analysis showed that CRC patient survival was less in high miR-27a-3p expression group compared with low miR-27a-3p expression group (p=0.047) (Figure 1C). Univariate Cox regression analysis showed that histological differentiation, Dukes staging, N classification, M classification, and miR-27a-3p expression were significantly associated with the prognosis of CRC patients. Multivariable Cox regression analysis showed that Dukes staging and miR-27a-3p expression were independent prognostic factors for CRC patients (Table 2–3).

**Table 1:** miR-27a-3p expression and its association with clinicopathological features of CRC

Characteristics	Case (No.)	Expression level of miR-27a-3p	*p* value
Gender			
Male	53	0.281±0.778	0.512
Female	47	0.183±0.705	
Age (Years)			
<60	42	0.260±0.742	0.778
≥60	58	0.217±0.749	
Tumor size			
<5cm	34	0.155±0.658	0.443
≥5cm	66	0.276±0.784	
Histological differentiation			
Well	23	-0.049±0.722	0.036
Moderately∼Poorly	77	0.320±0.732	
Dukes staging			
A	9	-0.220±0.828	0.045
B	45	0.304±0.681	
C	36	0.156±0.651	
D	10	0.622±1.058	
T classification			
T0∼T2	21	0.058±0.740	0.222
T3∼T4	79	0.282±0.741	
N classification			
N0	59	0.310±0.770	0.224
N1∼N2	41	0.126±0.696	
M classification			
M0	87	0.201±0.649	0.006
M1	13	0.778±0.978	

**Table 2:** Univariate Cox regression analysis for the prognostic value of miR-27a-3p expression and clinicopathological features in CRC

Characteristics	Case (No.)	Hazard ratio	95% CI	*p* value
Gender				
Male	53	1.110	0.521∼2.367	0.787
Female	47			
Age (Years)				
<60	42	0.886	0.415∼1.895	0.756
≥60	58			
Tumor size				
<5cm	34	1.702	0.719∼4.028	0.227
≥5cm	66			
Histological differentiation				
Well	23	9.574	1.298∼70.605	0.027
Moderately∼Poorly	77			
Dukes staging				
A	9	3.778	2.230∼6.403	<0.001
B	45			
C	36			
D	10			
T classification				
T0∼T2	21	2.053	0.618∼6.823	0.240
T3∼T4	79			
N classification				
N0	59	2.167	1.115∼4.208	0.022
N1∼N2	41			
M classification				
M0	87	5.791	2.554∼13.130	<0.001
M1	13			
MiR-27a-3p expression				
High	64	3.525	1.043∼11.919	0.043
Low	36			

**Table 3:** Multivariate Cox regression analysis for the prognostic value of miR-27a-3p expression and clinicopathological feature CRC

Characteristics		Case (No.)	Hazard ratio	95% CI	*p* value
Dukes staging	A	9	4.016	1.783∼9.043	0.001
	B	45			
	C	36			
	D	10			
MiR-27a-3p expression	High	64	3.628	1.030∼12.776	0.045
	Low	36			

### MiR-27a-3p promotes CRC growth, migration and invasion and suppresses apoptosis *in vitro* and *in vivo*

**Figure 2:**
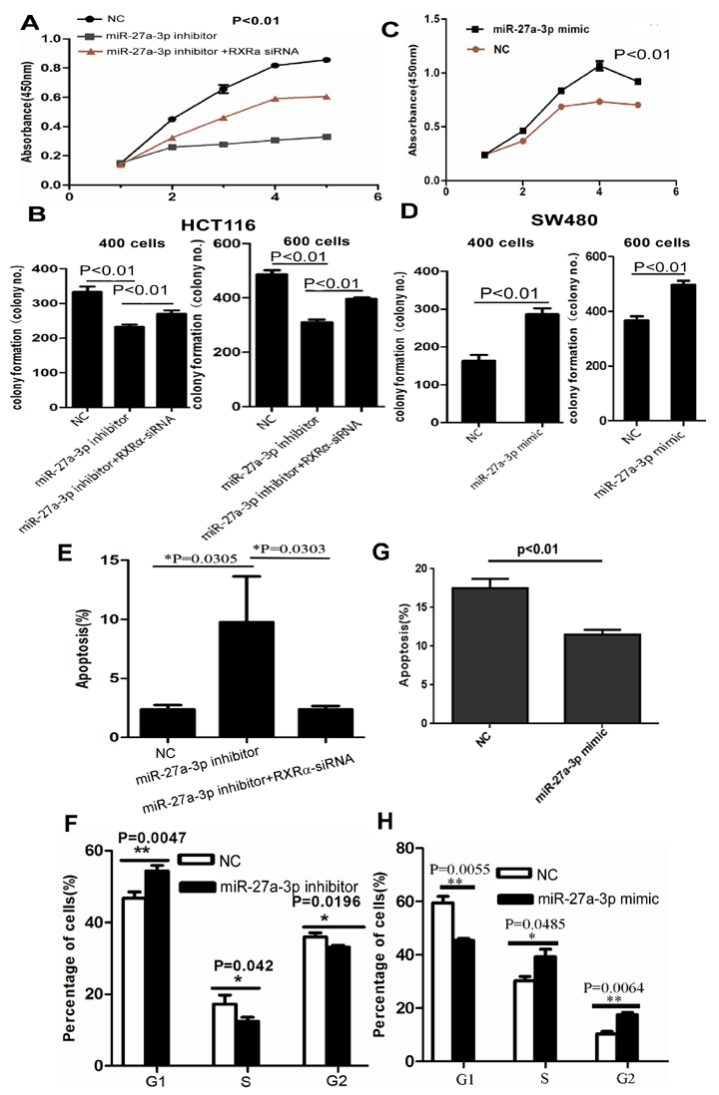
**(A-B)** miR-27a-3p inhibitor significantly suppressed cell proliferation (A) and clone formation (B) in HCT116. However, RXRα knockdown reversed the suppression of cell proliferation and clone formation in HCT116 induced by miR-27a-3p inhibitor. **(C-D)** miR-27a-3p mimic significantly increased cell proliferation (C) and clone formation (D) in SW480 compared with the control group. **(E-F)** miR-27a-3p inhibitor significantly induced cell apoptosis (E) and cell cycle G-S phase arrest (F) in HCT116. However, RXRα knockdown reversed elevated cell apoptosis in HCT116 induced by miR-27a-3p inhibitor. **(G-H)** miR-27a-3p mimic significantly suppressed cell apoptosis (G) and cell cycle G-S phase arrest (H) in SW480 compared with the control group.

**Figure 3:**
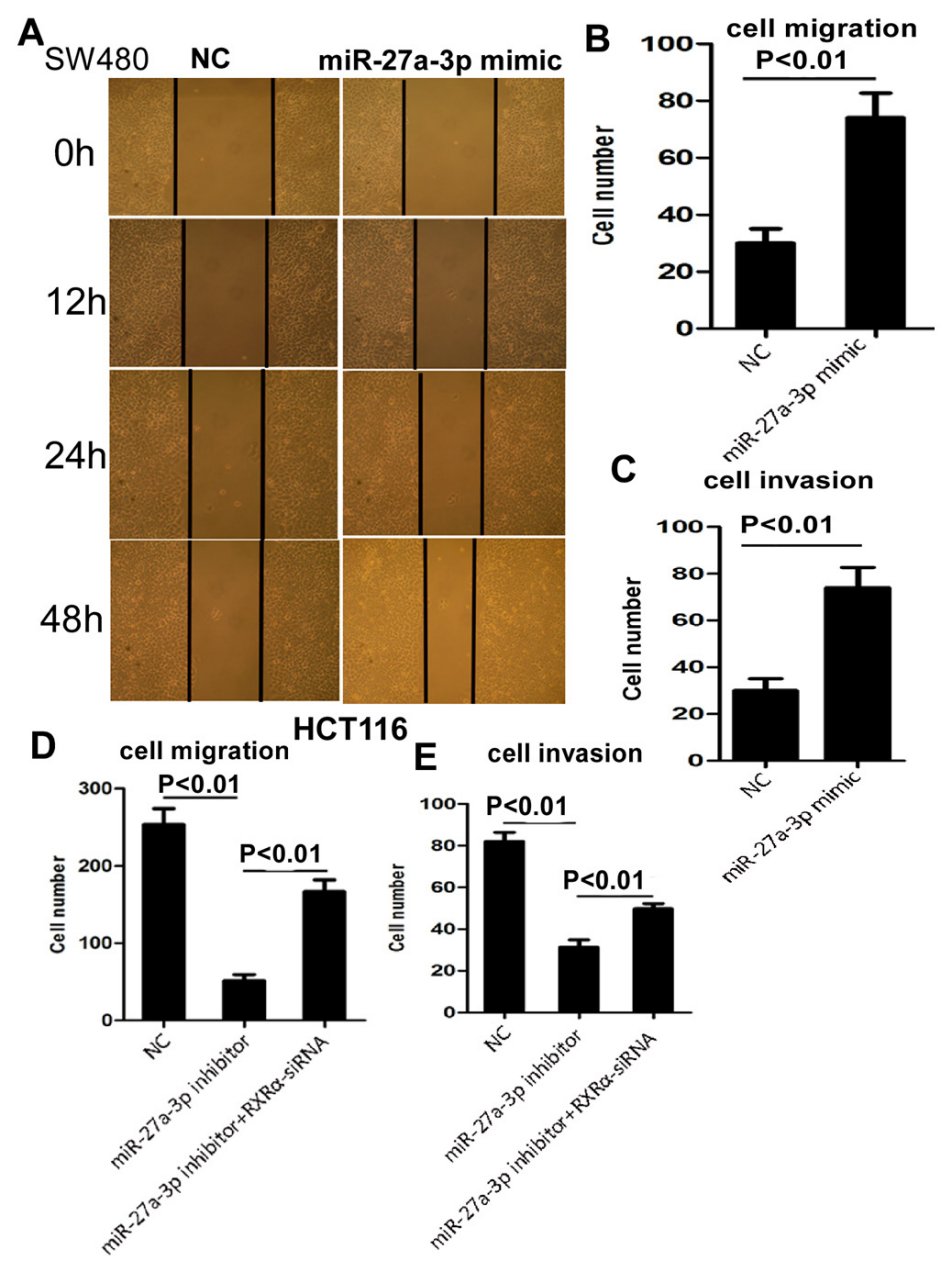
**(A-B)** miR-27a-3p mimic significantly increased SW480 cell migration by scratch wound healing assay (A) and cell migration assay (B), respectively. **(C)** miR-27a-3p mimic significantly increased SW480 cell invasion compared with the control group. **(D-E)** miR-27a-3p inhibitor significantly suppressed HCT116 cell migration (D) and invasion (E). However, RXRα knockdown reversed suppression of cell migration (D) and invasion (E) in HCT116 induced by miR-27a-3p inhibitor.

We then examined the biological function of miR-27a-3p in CRC cells. As shown in Figure 2A-2D, cell proliferation was suppressed significantly in HCT116 cells transfected with miR-27a-3p inhibitor and enhanced in SW480 cells transfected with miR-27a-3p mimic compared with the control group, respectively. In addition, miR-27a-3p inhibitor and mimic significantly suppressed and promoted clone formation in HCT116 and SW480 cells compared with the control group at 400 and 600 different cell densities, respectively. Flow cytometry analysis showed that elevated apoptosis and cell cycle G1-S phase arrest were found in HCT116 transfected with miR-27a-3p inhibitor compared with the control group, respectively. Decreased apoptosis and cell cycle G1-S phase arrest were found in SW480 transfected with miR-27a-3p mimic compared with the control group, respectively (Figure 2E–2H). miR-27a-3p mimic significantly increased SW480 cell migration by scratch wound healing assay and cell migration assay, respectively. Similarly, cellular invasion assay showed that miR-27a-3p mimic significantly increased SW480 cell invasion (Figure 3A–3C). miR-27a-3p inhibitor significantly suppressed HCT116 cell migration and invasion compared with the control group, respectively (Figure 3D–3E).

**Figure 4:**
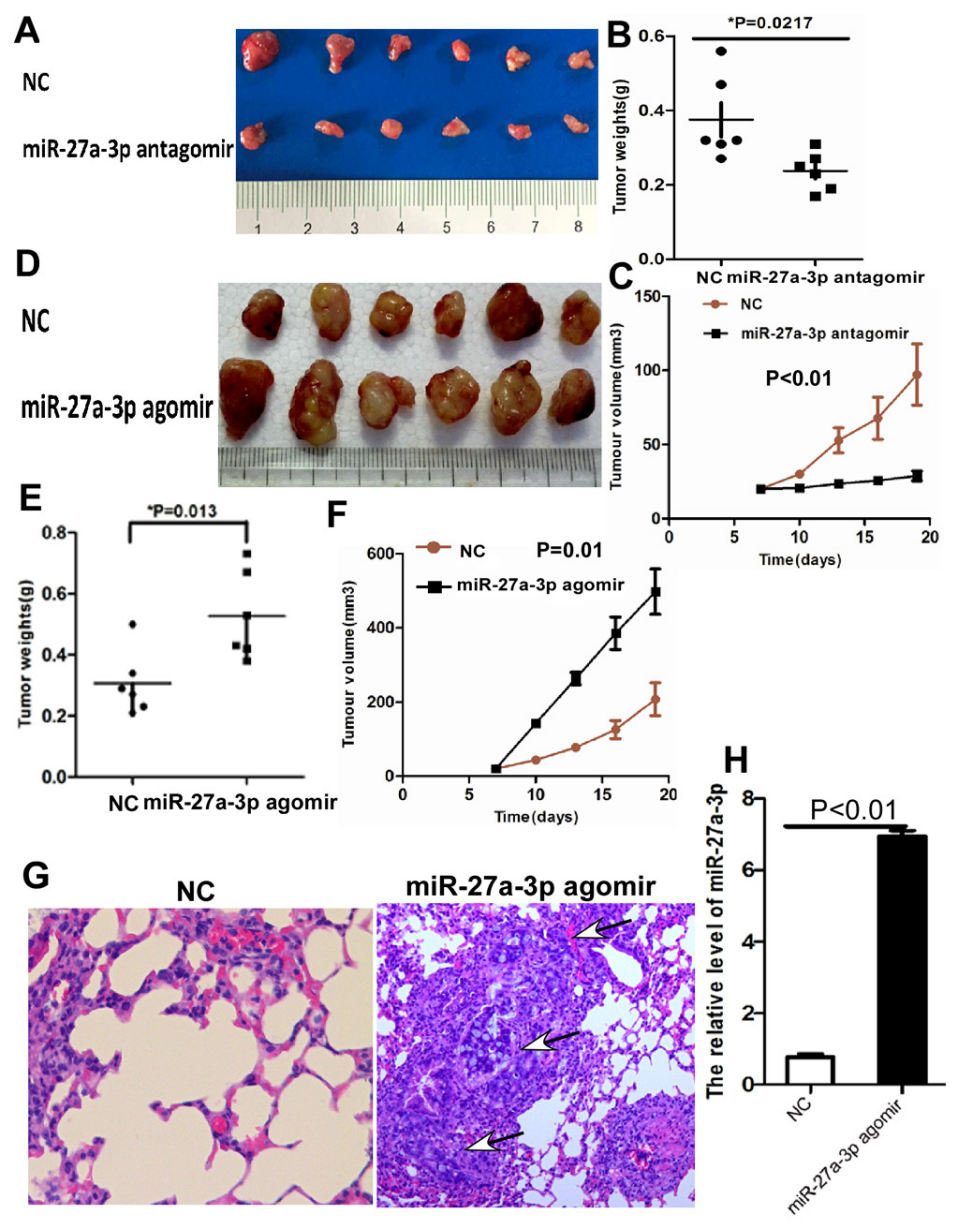
**(A-C)** miR-27a-3p antagomir significantly suppressed tumor growth (A), tumor weight (B), and tumor volume (C) of HCT116 cells implanted subcutaneously in BALB/c-nu mice compared with the control group, respectively. **(D-F)** miR-27a-3p agomir significantly increased tumor growth (D), tumor weight (E), and tumor volume (F) of SW480 cells implanted subcutaneously in BALB/c-nu mice compared with the control group, respectively. **(G)** Histological staining showed the lung metastatic carcinoma (arrow indicated) of tumor xenografts generated by HT29 cells transfected with miR-27a-3p agomir compared with the control group (NC), haematoxylin and eosin staining ×200. **(H)** miR-27-3p expression was significantly higher in tumor xenografts generated by HT29 cells transfected with miR-27a-3p agomir compared with the control group (NC) by quantitative real-time PCR.

To further investigate the role of miR-27a-3p on CRC growth and metastasis *in vivo*, we performed xenograft tumor assays using HCT116 and SW480 cells. As shown in Figure 4A-4C, xenograft tumor growth, weight, and volume were significantly inhibited in HCT116 treated with miR-27a-3p antagomir compared with the control group, respectively. However, xenograft tumor growth, weight, and volume significantly increased in SW480 treated with miR-27a-3p agomir compared with the control group, respectively (Figure 4D–4F). The effect of miR-27a-3p on CRC metastasis was performed in metastatic animal model using HT29 cells. The results showed that pulmonary metastatic CRC was found in 2 of 6 mice treated with miR-27a-3p agomir but no metastatic CRC tumor in the lung (0/6 mice) was found in the control group (Figure 4G). miR-27a-3p expression was significantly higher in pulmonary metastatic CRC tumor in treatment group than that in lung of control group by real-time PCR analysis (p<0.01, Figure 4H).

### RXRα is the target gene of miR-27a-3p and indispensable to miR-27a-3p-mediated oncogenic role in CRC

**Figure 5:**
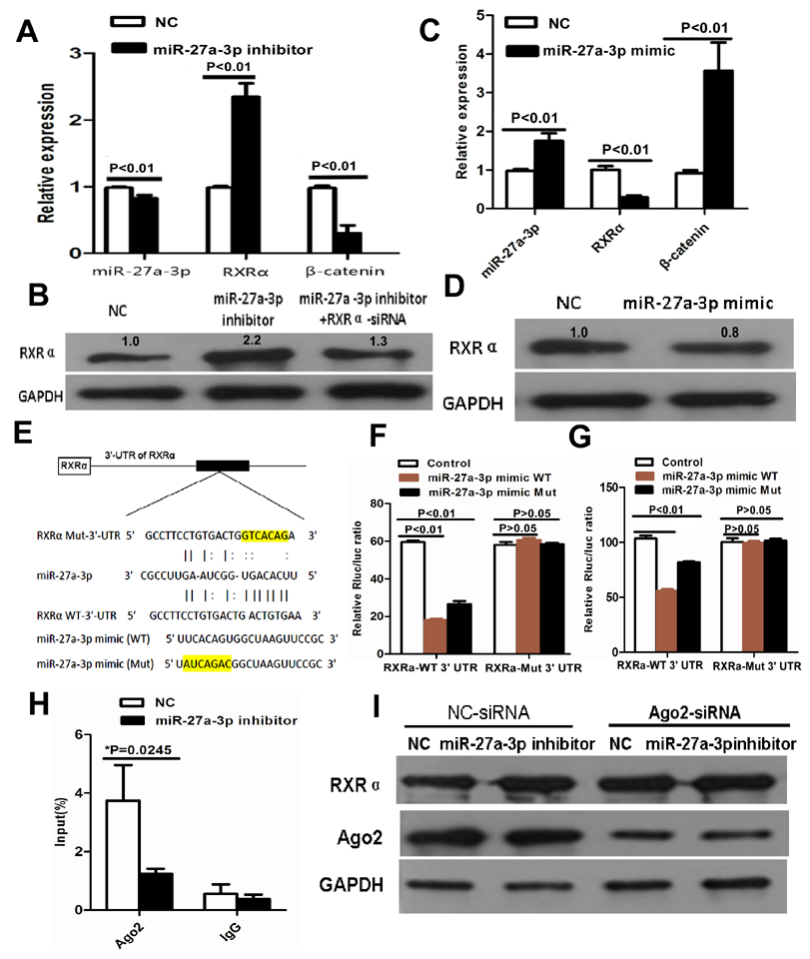
**(A)** miR-27a-3p inhibitor increased RXRα mRNA and suppressed β-catenin mRNA expression in HCT116 by real-time PCR. **(B)** miR-27a-3p inhibitor increased RXRα protein expression in HCT116, however, RXRα knockdown reversed elevated RXRα protein expression which was induced by miR-27a-3p inhibitor in HCT116 by western blot analysis. The relative quantification of bands in Western blots was a ratio neutralized to GAPDH. **(C)** miR-27a-3p mimic suppressed RXRα mRNA and increased β-catenin mRNA expression in SW480 by real-time PCR. **(D)** miR-27a-3p mimic suppressed RXRα protein expression in SW480 by western blot analysis. The relative quantification of bands in Western blots was a ratio neutralized to GAPDH. **(E)** Predicted binding sites of miR-27a-3p in the wild type 3’-UTR of RXRα. Mutations in the 3’-UTR of RXRα and miR-27a-3p mimic were highlighted in yellow. **(F-G)** miR-27a-3p significantly inhibited the luciferase activities of RXRα-WT 3’UTR reporter in HCT116 (F) and SW480 (G) cells. However, miR-27a-3p had no effect on the luciferase activities of RXRα-Mut 3’UTR reporter in HCT116 (F) and SW480 (G) cells. **(H)** RNA binding protein immunoprecipitation assay showed Ago2 was associated with miR-27a-3p. **(I)** Ago2 knockdown dramatically decreased the effect of miR-27a-3p on RXRα expression in HCT116 cells.

To clarify the molecular mechanism of miR-27a-3p in CRC progress, we predicted that miR-27a-3p UGACACU could completely bind ACTGTGA of RXRα mRNA 3’UTR using TargetScan, Diana Tools, PicTar, and miRanda software. First, we analyzed miR-27a-3p and RXRα expression in 100 samples of CRC tissues. As shown in Table 4, High RXRα expression was found in 33% (12/36) of miR-27a-3p low expression group, low RXRα expression was found in 86% (55/64) of miR-27a-3p high expression group. A significantly negative correlation between miR-27a-3p and RXRα expression was found in CRC tissues (r=-0.227, p=0.023). Further study showed that RXRα mRNA and protein expression was increased in HCT116 cells transfected with miR-27a-3p inhibitor compared with the control group, respectively. RXRα mRNA and protein expression was suppressed in SW480 cells transfected with miR-27a-3p mimic compared with the control group, respectively (Figure 5A–5D). To further investigate whether the predicted binding site of miR-27a-3p to the 3’UTR of RXRα was specific, we cloned the 3’UTR of RXRα (RXRα-WT) and its mutant variant (RXRα-Mut) into the downstream of luciferase reporter gene. Luciferase reporter assay showed that miR-27a-3p mimic-WT significantly suppressed luciferase activity of RXRα-WT 3’-UTR in SW480 and HCT116 compared with the control group, respectively. The suppressed luciferase activity of RXRα-WT 3’-UTR was reversed by miR-27a-3p mimic-Mut in HCT116 and SW480 cells, respectively. However, miR-27a-3p mimic-WT or miR-27a-3p mimic-Mut did not affect the luciferase activity of RXRα-Mut 3’-UTR in SW480 and HCT116 compared with the control group, respectively (Figure 5E–5G). We further performed RNA Binding Protein Immunoprecipitation to determine whether Ago2 was associated with RXRα transcripts regulated by miR-27a-3p. Our result showed that anti-Ago2 efficiently captured RXRα transcripts. miR-27a-3p inhibitor significantly reduced RXRα transcripts captured by anti-Ago2 (p=0.025). Further study showed that Ago2 knockdown dramatically decreased the effect of miR-27a-3p on RXRα expression in HCT116 cells (Figure 5H–5I).

**Table 4:** The correlation between miR-27a-3p expression and RXRα expression in CRC

Variable		miR-27a-3p expression level			
		Low (%)	High (%)	*P* value	r
**RXRα expression level**	Low	24(24%)	55(55%)	0.023	-0.227
	High	12(12%)	9(9%)		

Whether is RXRα indispensable to miR-27a-3p-mediated oncogenic role in CRC? The CCK8 assay (Figure 2A), colony formation assay (Figure 2B), and apoptosis assay (Figure 2E) showed that miR-27a-3p inhibitor significantly impaired HCT116 cell proliferation and colony formation, but resulted in more apoptosis, which could be reversed by RXRα knockdown. Likewise, RXRα knockdown significantly reversed the suppression of cell migration and invasion in HCT116 transfected with miR-27a-3p inhibitor, respectively (Figure 3D–3E).

### MiR-27a-3p/RXRα/Wnt/β-catenin signaling pathway is involved in CRC

**Figure 6:**
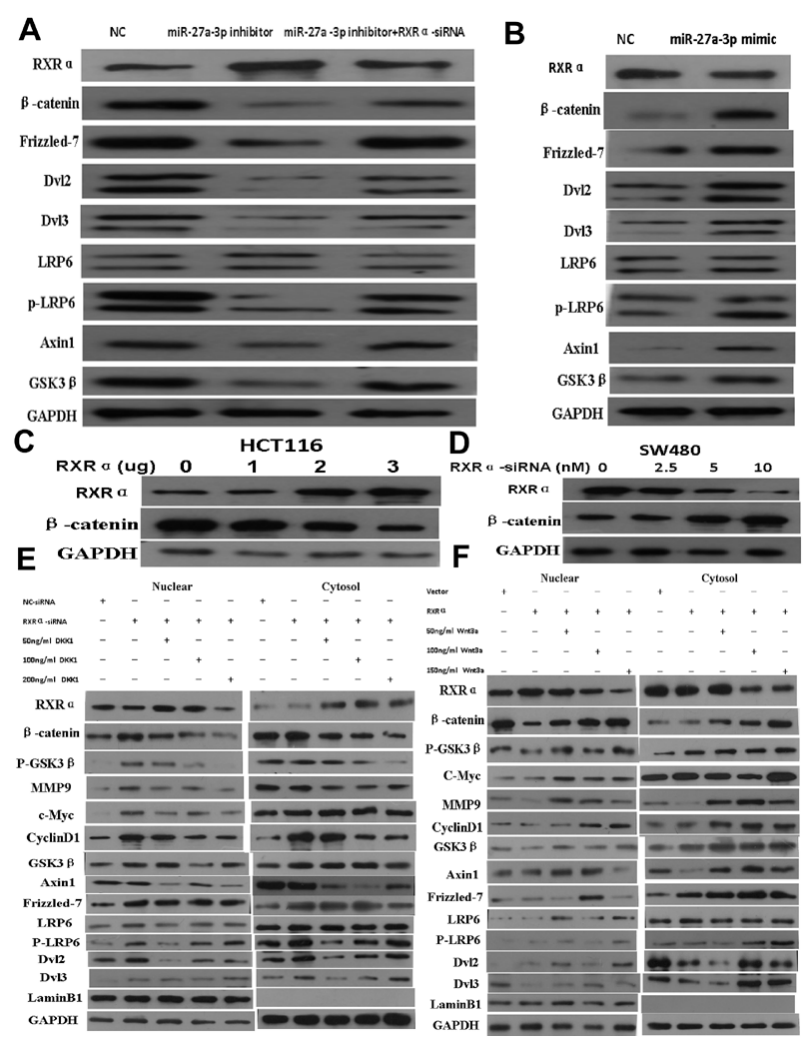
**(A)** miR-27a-3p inhibitor increased RXRα and suppressed β-catenin, Frizzled-7, Dvl2, Dvl3, p-LRP6, Axin1, and GSK3β expression in HCT116 cells. However, RXRα knockdown reversed the suppression of β-catenin, Frizzled-7, Dvl2, Dvl3, p-LRP6, Axin1, and GSK3β expression in HCT116 cells by miR-27a-3p inhibitor. **(B)** miR-27a-3p mimic suppressed RXRα expression and increased β-catenin, Frizzled-7, Dvl2, Dvl3, p-LRP6, Axin1, and GSK3β expression in SW480 cells. **(C)** Ectopic RXRα suppressed β-catenin expression in HCT116 cells. **(D)** RXRα knockdown resulted in increased β-catenin protein level in SW480. **(E)** DKK1 dramatically reversed increased β-catenin, p-GSK3β, MMP9, c-Myc, cyclinD1, Axin1, Frizzled-7, p-LRP6, Dvl2, and Dvl3 in HCT116 with RXRα knockdown. **(F)** Wnt3a dramatically reversed suppressed β-catenin, p-GSK3β, MMP9, c-Myc, cyclinD1, Axin1, Frizzled-7, p-LRP6, Dvl2, and Dvl3 in SW480 transfected with RXRα expression plasmid.

Whether Wnt/β-catenin signaling pathway is involved in the mechanism of miR-27a-3p targeting RXRα in CRC progression, our data showed that miR-27a-3p inhibitor dramatically increased RXRα and suppressed β-catenin, Frizzled-7, Dvl2, Dvl3, p-LRP6, Axin1, and GSK3β expression in HCT116 cells compared with the control group. However, RXRα knockdown reversed the suppression of β-catenin, Frizzled-7, Dvl2, Dvl3, p-LRP6, Axin1, and GSK3β expression in HCT116 cells by miR-27a-3p inhibitor. miR-27a-3p mimic dramatically suppressed RXRα but increased β-catenin, Frizzled-7, Dvl2, Dvl3, p-LRP6, Axin1, and GSK3β expression in SW480 cells compared with the control group (Figure 6A–6B).

**Figure 7:**
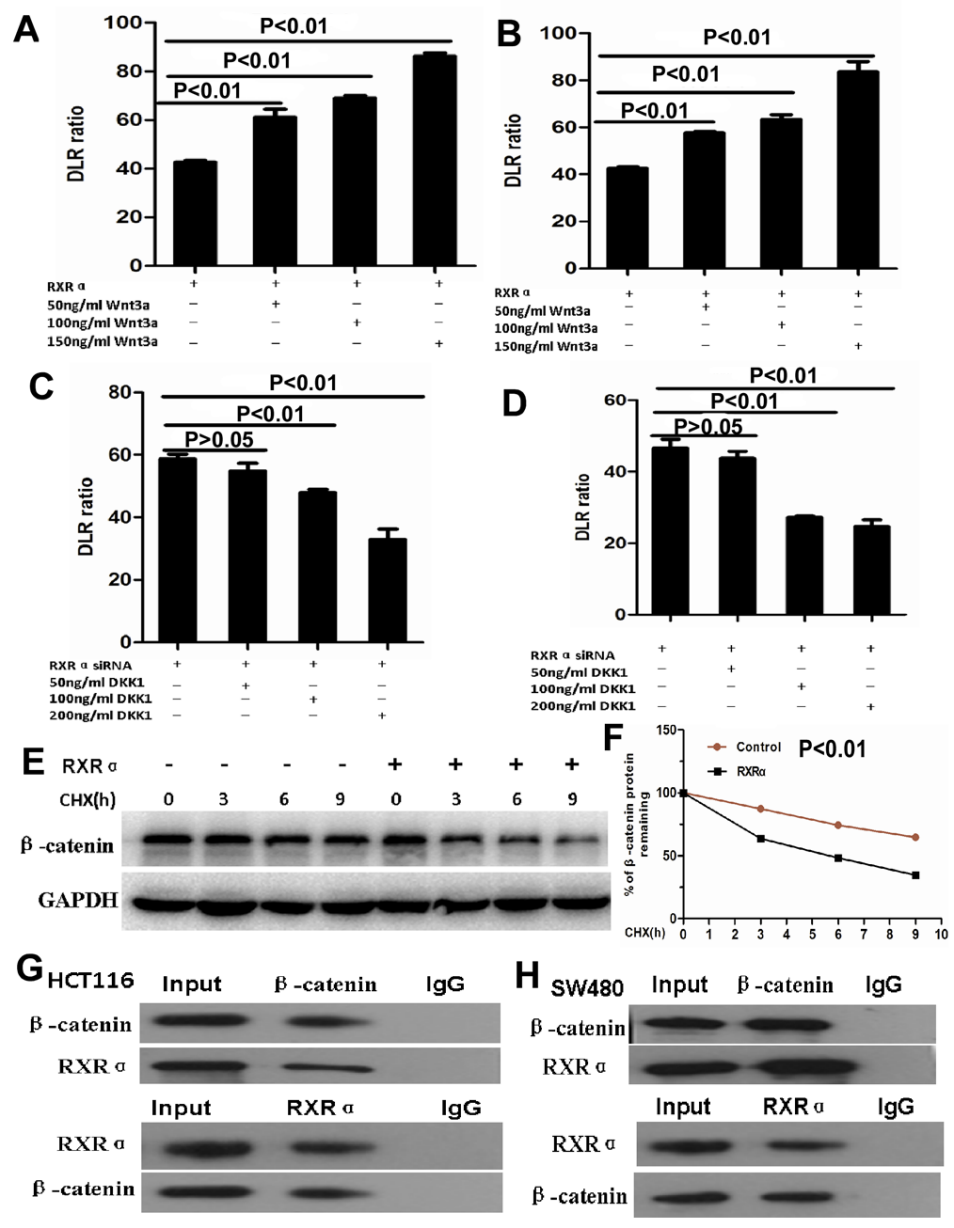
**(A-B)** TCF/LEF Luciferase reporter assay showed Wnt3a significantly reversed suppressed β-catenin transcriptional activity in HCT116 (A) and SW480 (B) cells transfected with RXRα expression plasmid and in a dose dependent manner. **(C-D)** DKK1 significantly reversed increased β-catenin transcriptional activity in HCT116 (C) and SW480 (D) cells transfected with RXRα siRNA and in a dose dependent manner. **(E-F)** the half-life of β-catenin protein was shortened significantly in HCT116 cells transfected with RXRα and treated with cycloheximide compared with the control group. **(G-H)** Co- immunoprecipitation showed that RXRα interacted with β-catenin in HCT116 (G) and SW480 (H).

Further study showed that β-catenin expression was suppressed proportionally with progressively increasing amounts of ectopic RXRα in HCT116. RXRα knockdown resulted in increased β-catenin protein expression in SW480 (Figure 6C–6D). DKK1 dramatically reversed increased β-catenin and its signaling pathway proteins including p-GSK3β, MMP9, c-Myc, cyclinD1, Axin1, Frizzled-7, p-LRP6, Dvl2, and Dvl3 in HCT116 with RXRα knockdown. Likewise, Wnt3a dramatically reversed suppressed β-catenin and its signaling pathway proteins including p-GSK3β, MMP9, c-Myc, cyclinD1, Axin1, Frizzled-7, p-LRP6, Dvl2, and Dvl3 in SW480 transfected with RXRα expression plasmid (Figure 6E–6F). TCF/LEF Luciferase reporter assay showed Wnt3a significantly reversed suppressed β-catenin transcriptional activity in HCT116 and SW480 cells transfected with RXRα expression plasmid and in a dose dependent manner, respectively (Figure 7A–7B). DKK1 significantly reversed increased β-catenin transcriptional activity in HCT116 and SW480 cells transfected with RXRα siRNA and in a dose dependent manner, respectively (Figure 7C–7D). The half-life of β-catenin protein was shortened significantly in HCT116 cells transfected with RXRα and treated with cycloheximide compared with the control group (Figure 7E–7F). Furthermore, a direct interaction between RXRα and β-catenin was found in HCT116 and SW480 by co-immunoprecipitation assay (Figure 7G–7H).

**Figure 8:**
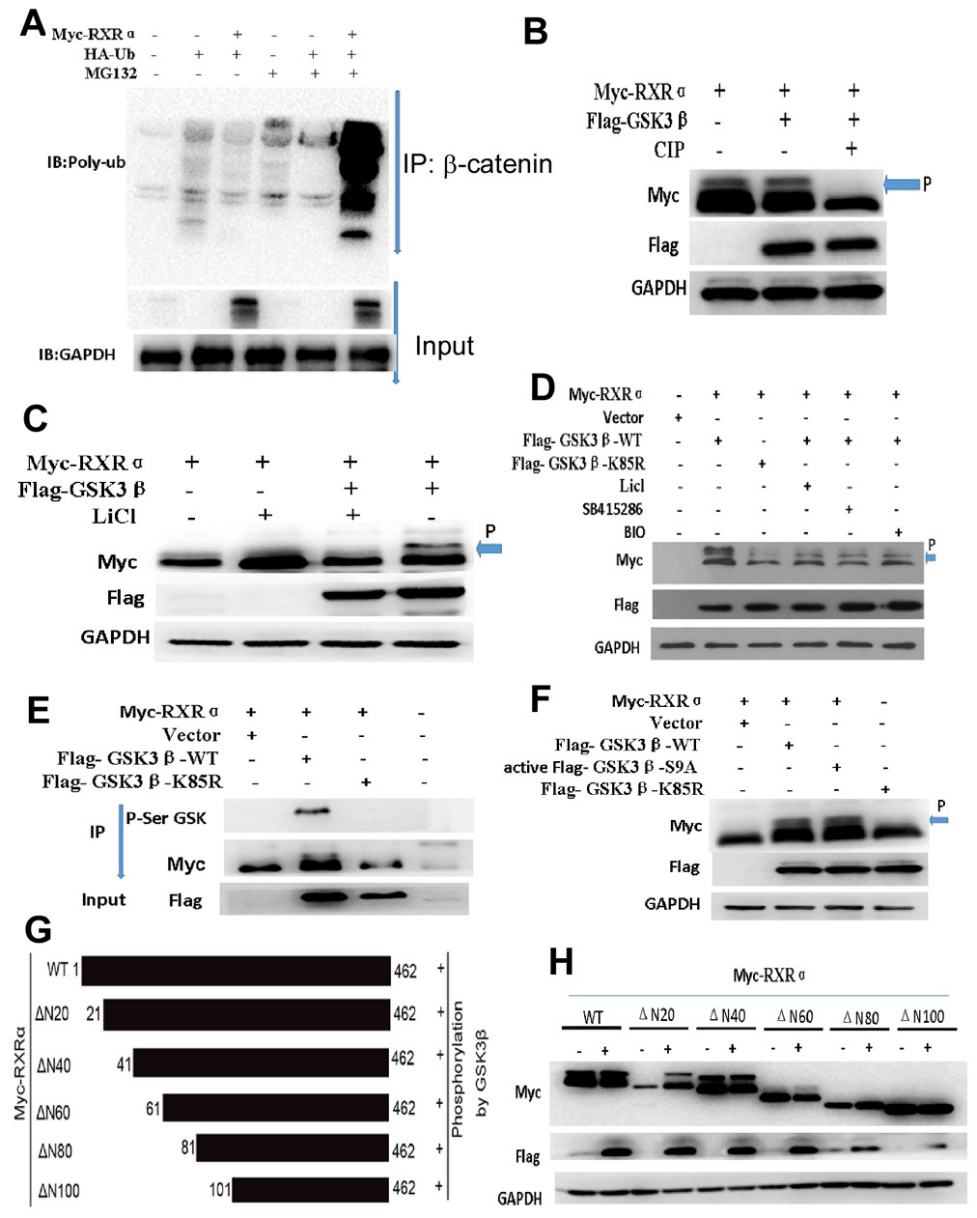
**(A)** Immunoblotting showed that the ubiquitination of β-catenin was dramatically augmented by RXRα overexpression in HCT116 cells treated with MG132. **(B)** phosphorylation inhibitor-CIP impaired the effect of GSK3β phosphorylation on RXRα by western blot (arrow and p indicated phosphorelated RXRα). **(C-D)** LiCl, BIO, and SB415286 as inhibitors of GSK3β could suppress RXRα phosphorylation by GSK3β in HEK293T (C) and HCT116 (D) cells. **(E)** co-immunoprecipitation assay showed that there was a direct interaction between RXRα and GSK3β through GSK3β serine sites. **(F)** mutant-Flag-GSK3β-K85R impaired the effect of RXRα binding to GSK3β serine sites. GSK3β kinase-activated mutant plasmid-GSK3β-S9A could induce the phosphorylation of RXRα in HEK293T cells. **(G)** The schematic diagram depicts potential RXRα phosphorylation regions and various RXRα gene deletion mutants were constructed. **(H)** Western blot showed that deletion mutants of the N20 (ΔN20), N40 (ΔN40), and N60 (ΔN60) induced RXRα phosphorylation by GSK3β, respectively. However, deletion mutants of the N80 (ΔN80) and N100 (ΔN100) had no effect on GSK3β-induced RXRα phosphorylation in HEK293T cells.

Immunoblotting showed that the ubiquitination of β-catenin was dramatically augmented by RXRα overexpression in HCT116 cells treated with MG132 compared with the control group (Figure 8A). RXRα could be regulated by several protein kinases. To determine whether GSK3β phosphorylates RXRα and induces activation of Wnt/β-catenin pathway. Myc-RXRα and Flag-GSK3β plasmids were cotransfected in HCT116 cells. The anti-Myc antibody recognized the migrating upward band of RXRα, which stands for the phosphorylated RXRα. Phosphorylation inhibitor-calf intestinal alkaline phosphatase (CIP) impaired RXRα phosphorylation by GSK3β. Further study showed that LiCl, 6-bromoindir-ubin-30-oxime (BIO), and SB415286, which are inhibitors of GSK3β, suppressed RXRα phosphorylation by GSK3β in HEK293T and HCT116, respectively. Co-immunoprecipitation assay showed that there was a direct interaction between RXRα and GSK3β through GSK3β serine sites in HEK293 cells. However, transfection of mutant-Flag-GSK3β-K85R impaired the effect of RXRα binding to GSK3β serine sites. Moreover, GSK3β kinase-activated mutant plasmid-GSK3β-S9A induced RXRα phosphorylation in HEK293T cells (Figure 8B–8F).

**Figure 9:**
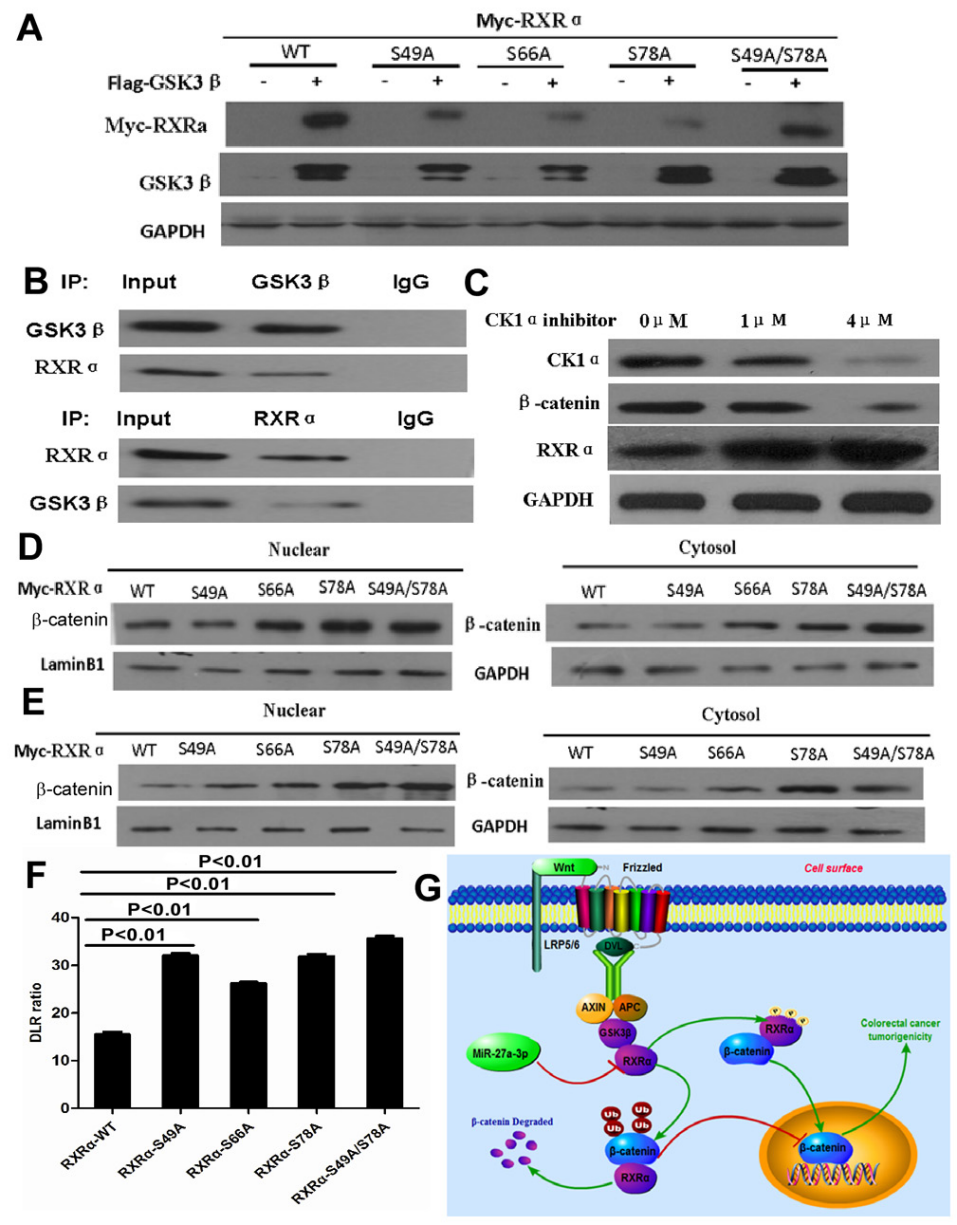
**(A)** GSK3β-induced RXRα phosphorylation decreased for RXRα-S49A, RXRα-S66A and RXRα-S78A in HEK293 cells compared with RXRα WT by western blot analysis. **(B)** The physical interaction of endogenous RXRα and GSK3β was found in HCT116 by co-immunoprecipitation. **(C)** Casein kinase 1α inhibitor increased RXRα expression and suppressed β-catenin expression in HCT116 and in a dose-dependent manner. **(D-E)** RXRα-S49A, RXRα -S66A and RXRα-S78A increased the nuclear β-catenin expression in HCT116 (D) and SW480 (E) cells compared with the stimulated p-Ser of RXRα WT by Western blot. **(F)** RXRα-S49A, RXRα-S66A and RXRα-S78A increased the TCF/LEF luciferase activity in HCT116 cells compared with RXRα WT, respectively. **(G)** Illustration of Wnt/β-catenin activation by miR-27a-3p targeting RXRα in CRC.

Next, we examined potential p-Ser sites of the RXRα protein using the Phosphor motif Finder program and identified some hypothetical p-Ser sites, which mainly located within N-domain 100 amino acids, a residue in RXRα predicted to be a target of Ser and other kinases. To determine whether GSK3β phosphorylates RXRα through one or more specific p-Ser sites of RXRα, we constructed various RXRα deletion mutants and cotransfected these various DNAs and Flag-GSK3β into human HEK293T cells. Our result showed that deletion mutants of the N20 (ΔN20), N40 (ΔN40), and N60 (ΔN60) induced RXRα phosphorylation, respectively. However, deletion mutants of the N80 (ΔN80) and N100 (ΔN100) had no effect on GSK3β-induced RXRα phosphorylation (Figure 8G–8H). Three p-Ser candidate sites at Ser49, Ser66 and Ser78 were identified within the targeted domain of RXRα. To ascertain that Ser49, Ser66, and Ser78 are major p-Ser sites of RXRα induced by GSK3β, Ser49, Ser66 and Ser78 were mutated to Ala (A) in the full-length RXRα protein, respectively. GSK3β-induced p-Ser was markedly reduced for RXRα^S49A^, RXRα^S66A^and RXRα^S78A^ in HEK293 cells compared with the stimulated p-Ser of RXRα^WT^ by western blot analysis. The physical interaction of endogenous RXRα and GSK3β was found in HCT116 by co- immunoprecipitation assay. Since casein kinase 1α (CK1α) mediates GSK3β-induced p-Ser of protein, our result showed that CK1α inhibitor increased RXRα expression and suppressed β-catenin expression in HCT116 and in a dose-dependent manner (Figure 9A–9C).

To further determine whether p-RXRαS49, p-RXRαS66 and p-RXRαS78 induced by GSK3β plays a role in Wnt/β-catenin signaling pathway in CRC. Our result showed that RXRα-S49A, RXRα-S66A, and RXRα-S78A increased the nuclear and cytoplasm β-catenin expression in HCT116 and SW480 cells compared with the stimulated p-Ser of RXRα WT, respectively. Moreover, β-catenin transcriptional activity was significantly enhanced in HCT116 transfected with RXRα-S49A, RXRα-S66A, and RXRα-S78A plasmids in HCT116 compared with RXRα-WT plasmid group by TCF/LEF luciferase reporter assay (Figure 9D–9F).

## DISCUSSION

CRC is one of the frequently seen malignancies in the world. Kara et al. have analyzed a total of 54 CRC and normal colon tissue samples of 42 healthy controls using a high-throughput real-time PCR method and found that miR-27a-3p was significantly deregulated in CRC [21]. Large-scale microRNA expression profiling was performed in serum samples from 427 CRC patients and 276 healthy donors using Illumina small RNA sequencing and a diagnostic four-microRNA signature consisting of miR-23a-3p, miR-27a-3p, miR-142-5p and miR-376c-3p was established [11]. miR-27a-3p was an independent predictive factor for recurrence in clear cell renal cell carcinoma [15]. However, expression of miR-27a-3p was consistently down-regulated in hepatocellular carcinoma cell lines and tissue samples and suppressed tumor metastasis [13]. Expression of microRNA-27a was increased in CRC stem cells [17]. The miR-27a- calreticulin axis affects drug-induced immunogenic cell death in human colorectal cancer cells [18]. Our data showed that expression of miR-27a-3p was up-regulated in CRC cell lines and tissues and closely associated with histological differentiation, clinical stage, distant metastasis and CRC patients’ survival. miR-27a-3p expression was an independent prognostic factor for CRC patients. However, Bao et al. have reported microRNA-27a is a tumor suppressor in colorectal carcinogenesis and progression by targeting SGPP1 and Smad2 [19]. Our result was different from Bao’s report, the reason includes: 1) we studied miR-27a-3p, Bao et al. studied microRNA-27a, whether microRNA-27a-3p and/or microRNA-27a-5p was studied is not clear. 2) we performed miR-27a-3p expression in large number of CRC with 100 samples, Bao et al. only used 41 CRC samples to detect microRNA-27a expression. This issue needs further study in larger samples of CRC.

As for the role of miR-27a-3p in malignant tumors, overexpression of miR-27a-3p promoted gastric cancer cell proliferation *in vitro* as well as tumor growth *in vivo* [12]. High expression of miR-27a enhances proliferation and invasion of CRC cells [22]. Consistent with other reports, our data showed that miR-27a-3p mimic suppressed apoptosis and promoted proliferation, migration, invasion of CRC cells *in vitro* and *in vivo*, whereas miR-27a-3p inhibitor suppressed CRC progression. The results suggest that miR-27a-3p plays an important role in promoting carcinogenesis of CRC.

Our previous study found that suppression of RXRα and aberrant β-catenin expression significantly associated with progression of CRC [10]. Methylation of RXRα might be related to RXRα expression suppression in CRC [23]. Pro-apoptotic miRNA-128-2 modulates ABCA1, ABCG1 and RXRα expression and cholesterol homeostasis. MicroRNA-27a contributes to rhabdomyosarcoma cell proliferation by suppressing RARA and RXRα[20]. Our data first showed that miR-27a-3p expression negatively correlated with RXRα expression in CRC tissues. RXRα was the target gene of miR-27a-3p in CRC and indispensable to miR-27a-3p-mediated oncogenic role in CRC. miR-27a could reverse multiple drug resistance in hepatocellular carcinoma cells by inhibiting the FZD7/β-catenin pathway [24]. MiR-27a-3p modulates the Wnt/β-catenin signaling pathway to promote epithelial- mesenchymal transition in oral squamous carcinoma stem cells by targeting SFRP1 [16]. Our previous study found that RXRα directly interacted with β-catenin and suppresses β-catenin transcription activity and protein expression in CRC cells [25]. Our data showed that RXRα was indispensable to miR-27a-3p-mediated oncogenic role in CRC. To further study the underlining mechanism of miR-27a-3p promoting carcinogenesis of CRC by targeting RXRα, our data demonstrated that miR-27a-3p inhibitor dramatically increased RXRα and suppressed β-catenin, Frizzled-7, Dvl2, Dvl3, p-LRP6, Axin1, and GSK3β expression in CRC cells. However, RXRα knockdown reversed the suppression of β-catenin, Frizzled-7, Dvl2, Dvl3, p-LRP6, Axin1, and GSK3β expression in CRC cells by miR-27a-3p inhibitor. The results suggest that miR-27a-3p/RXRα/Wnt/β-catenin signaling pathway is involved in carcinogenesis of CRC. GSK-3β plays an important role in regulating tRXRα production by calpain II in cancer cells [26]. Whether RXRα could be regulated by GSK3β in CRC? Our study showed that RXRα negatively regulated β-catenin expression by ubiquitination of β-catenin in CRC. Moreover, Ser49, Ser66, and Ser78 of RXRα, which are the three major p-Ser sites, were specifically phosphorylated by GSK3β in CRC cells. p-RXRαS49, p-RXRαS66, and p-RXRαS78 were important for activation of Wnt/β-catenin pathway in CRC cells. microRNA-224, retinoic acid receptor gamma, epigenetic silencing of miR-490-3p, and inhibition of long non-coding RNA-CTD903 promotes aggressive CRC phenotype by activation of the Wnt/β-catenin signaling pathway [27–30]. miR-27a-3p suppresses tumor metastasis and vasculogenic mimicry by down-regulating vascular endothelial -cadherin expression and inhibiting EMT: an essential role for Twist-1 in HCC [13]. Whether there are other signaling pathway of miR-27a-3p-mediated oncogenic role in CRC needs further study.

In conclusion, our findings first demonstrate that miR-27a-3p is a prognostic and/or potential therapeutic biomarker for CRC patients and RXRα as miR-27a-3p targeting gene plays the critical role in activation of Wnt/β-catenin pathway during CRC progression.

## MATERIALS AND METHODS

### Clinical samples and patient information

100 pairs of paraffin-embedded archived CRC and adjacent non-tumor colorectal mucosal tissues (ANT) were collected from our Department between January 2000 and December 2013. Fifteen pairs of fresh CRC and ANT were collected in 2013. No patients had received chemotherapy and/or radiotherapy before operation. The histopathology of the disease was determined by two pathologists according to the criteria of the World Health Organization. Clinical staging was done according to Dukes staging. For the research purposes of these clinical materials, prior patient’s consents and approval from the Institutional Research Ethics Committee were obtained. Detailed clinical information about these patients, including age, gender, clinical stage, histological differentiation, T classification, N classification, and distant metastasis status, is summarized in Table 1. Follow-up information was available for all patients.

### Cell lines and small interfering RNA (siRNA) sequences

The human CRC cell lines SW480 and SW620 were maintained in Leibovitz’s L-15 Medium (Invitrogen, Carlsbad, CA). HCT116 was grown in McCoy’s 5A Medium (Invitrogen). LOVO and SW1116 were cultured in RPMI-1640 medium (Invitrogen). HT29 was maintained in Dulbecco’s modified Eagle’s medium (Invitrogen). The human colonic epithelial cell line NCM460 was cultured in RPMI-1640 medium. All lines were purchased from Cell bank of Chinese Academy of Science (Shanghai, China) and authenticated by their karyotypes, and detailed gene expression in 2015. All medium were supplemented with 10% (v/v) fetal bovine serum (Invitrogen), 1×antibiotic/antimycotic (100units/mL streptomycin, 100units/mL penicillin, and 0.25 mg/ mL amphotericin B). All cell lines were cultured in humidified incubator at 37°C with 5% CO_2_.

The small interfering RNA (siRNA) specifically for RXRα and Ago2 were chemically synthesized and purified from Ribobio Inc. (Guangzhou, China). The sequences were: RXRα siRNA: sense 5’-AAG CAC UAU GGA GUG UAC AGC dTdT-3’; Ago2 siRNA: sense 5’-GCA CGG AAG UCC AUC UGAA dTdT-3’. The siRNA was transfected using Lipofectamine RNAiMAX transfection reagent (Invitrogen) according to the manufacturer’s instructions. Scrambled siRNA were used as negative control group. The human RXRα expression plasmid and GSK3β expression plasmid were purchased from Sino Biological Inc (Beijing, China).

### Cell proliferation assay

HCT116 and SW480 cells (2×10^3^) were plated onto 96-well plates with medium containing 10% FBS and incubated overnight. After transfection with miR-27a-3p mimic or inhibitor or RXRα-siRNA, cell proliferation was determined at 0, 24, 48, 72, and 96h using the Cell Counting Kit-8 (CCK8) (Keygene, China). The absorbance (OD) was measured at a wavelength of 450nm using a Microplate Autoreader (BioTek Instruments, USA). This experiment was performed in triplicate.

### Analysis of cell cycle and apoptosis

5 × 10^5^ cells were seeded in 6-well plates and incubated overnight until 50–60% confluence. The cells were transfected with 100nM miR-27a-3p mimic or inhibitor or RXRα-siRNA and harvested at 48h, washed in cold PBS, fixed with 80% ethanol for 8 h at 4°C, then stained with propidium iodide buffer (50mg/ml propidium iodide, 0.1% sodium citrate and 0.1% Triton X-100) for 3h at 4°C. Non-specific control miRNA mimic or inhibitor or scrambled-siRNA was used as the control group, respectively. 2× 10^4^ cells were analysed for cell cycle and apoptosis using a Becton Dickinson FACScan (Becton Dickinson Immunocytometry Systems, San Jose, CA). The percentage of cells in each phase of the cell cycle and apoptotic cells was quantified using Cell Quest software. This experiment was performed in triplicate.

### Colony formation assay

After 48h transfection with miR-27a-3p mimic or inhibitor or RXRα-siRNA, the cells at 4× 10^2^ and 6× 10^2^ per well were plated in 6-well plates and grown for 2 weeks, respectively. Then, the cells were washed twice with phosphate buffer saline (PBS), fixed with 4% paraformaldehyde and stained with 0.5% crystal violet for 15 min. The number of colonies in 10 random view fields was counted under a microscope and the average number of colonies was achieved. The experiment was triplicated independently.

### Scratch wound migration assay

2× 10^5^ SW480 cells were seeded in 6-well plates and grown to 60% confluency in complete medium. The cells were transfected with 100nM miR-27a-3p mimic. Non-specific control miRNA mimic was used as the control group. After 24h transfection, vertical scratches were then made using a 100μl plastic filter tip to create a ‘wound’ of approximately 100μm in a diameter. To eliminate dislodged cells, culture medium was removed and wells were washed with PBS. The average distance of migration cells was determined under an inverted microscope at 0, 12, 24 and 48h. The experiment was performed in triplicate.

### Transwell migration and invasion assays

Migration and invasion assays were carried out in Transwell chambers containing polycarbonate filters (8μm pore size; Corning Incorporated, Life Sciences, NY, USA). After transfected with 100nM miR-27a-3p mimic or inhibitor or RXRα-siRNA for 48h, HCT116 cells and SW480 cells (migration/2×10^4^ cells; invasion/2×10^5^ cells) in a 500μl volume of serum-free medium were placed in the upper chambers and incubated at 37°C with 5% CO_2_ for 24 hours, while a 200μl volume of medium containing 15%FBS was added to the lower chamber as chemoattractant. Cells were allowed to invade through the matrigel (BD Biosciences) or migrate for 24 hours at 37°C with 5% CO_2_. Following invasion or migration, cells were fixed with 4% formaldehyde and stained with 1% crystal violet. Cells on the upper surface of the filters were removed by wiping with a cotton swab. Cells counts were the mean number of cells per fields of view. Three independent experiments were performed and the data were presented as mean ± standard deviation (SD).

### Quantitative real time PCR

Reverse transcription was performed using One step PrimeScript miRNA and mRNA cDNA Synthesis Kit (Takara Biotechnology Co.Ltd, Dalian, China), and quantitative real-time PCR was performed using SYBR Premix Ex Taq II (Takara Biotechnology). RNAU6B snRNA and GAPDH was used for sample loading normalisation. The specific forward primer of miR-27a-3p was 5 ‘-ATG GTT CGT GGG TTC ACA GTG GCT AAG TTC CG-3’. The specific forward primer of RNAU6B was 5 ‘- ACG CAA ATT CGT GAA GCG TT-3’. Reverse primer for miR-27a-3p, U6B snRNA was Uni-miR qPCR primer (TakaRa code D350A). The primer sequences used for β-catenin were followed: forward: 5′-TTG AAA ATC CAG CGT GGA CA-3′; reverse: 5′-TCG AGT CAT TGC ATA CTG TC-3′. The primer sequences used for RXRα were followed: forward: 5’-GCA AGC TGG TGT GTC ATC AGC AAA-3′; reverse: 5’- ACA GAG GGC AGC TCA TGT TCT CAT-3′.

The quantity of miR-27a-3p in each CRC tissues relative to its paired ANT was calculated using the equation [RQ = 2^–ΔΔCT^, ΔΔCT= (CTmiRNA-CTU6)T – (CTmiRNA-CTU6)N]. The expression level of miR-27a-3p was classified into low expression and high expression group compared with RQ ratio =RQ(CRC)/RQ(ANT). The geometric mean of housekeeping gene GAPDH was used to normalize the variability at mRNA expression levels. All experiments were performed in triplicate.

### Western blot analysis

As we previously described [10], primary antibodies including RXRα (Abcam), Frizzled-7(Millipore), CK1α(Santa Cruz), β-catenin, GSK3β, LRP6, phosphorylated-LRP6 (Ser1490), Dvl2, Dvl3, Axin1 (Cell Signaling Technology, Danvers, MA) were used. Signal was detected by enhanced chemoluminescence techniques (Millipore). GAPDH or Lamin B1 (Cell Signaling Technology) was used as the loading control. After washing, the membranes were incubated with secondary antibody HRP-conjugated goat anti-rabbit (Cell Signalling Technology) for 1h at room temperature and visualised by enhanced chemiluminescence detection kit (Millipore).

### Immunohistochemistry staining

The working concentrations of primary antibody for the detection of RXRα (Santa Cruz Biotechnology, Santa Cruz, CA) was 1:200. Immunohistochemical staining was independently assessed by two observers who had no knowledge of the clinicopathologic data. The degree of RXRα immunostaining was scored as our previously reported [10].

### Construction of 3’-UTR-dual-luciferase plasmid and reporter assays

The miR-27a-3p sequence was obtained from miR-Base (http://www.microrna.sanger.ac.uk) and the target gene was predicted using bioinformatics software including Targetscan (www.targetscan.org). A fragment of 3’-UTR of RXRα (957bp) containing the putative miR-27a-3p binding site was amplified by PCR. The human RXRα-WT 3’-UTR plasmid was constructed by PCR amplification using primers as followed: forward: 5’-GTA CAG AGA GAC GCG TGT GG-3’; Reverse: 5’-CCT GAG GCC ACG ATG TTT CA-3’. The human RXRα-Mut 3’-UTR plasmid was constructed by PCR amplification using primers as followed: forward: 5’-GTA CAG AGA GAC GCG TGT GG-3’;Reverse:5’-CCT GAG GCC ACG ATG TTT CAG AGA CAA TCG TAC GGA GAA GCC ACC CTT TTC CCA GTC ACG GGA AGG CCA GAG-3’. The PCR product was subcloned into a pmiR-RB-REPORT™ vector immediately downstream to the luciferase gene sequence. A pmiR-RB-REPORT™ construct containing 3’-UTR of RXRα with a mutant sequence of miR-27a-3p was also synthesized. The miR-27a-3p mimic-WT and mimic-Mut sequences were 5’-UUC ACA GUG GCU AAG UUC CGC-3’ and 5’-UAU CAG ACG GCU AAG UUC CGC-3’, respectively. All constructs were verified by DNA sequencing. HCT116 and SW480 cells were plated in 96-well clusters, and cotransfected with 100ng constructs with miR-27a-3p mimic-WT or miR-27a-3p mimic-Mut (50nM/well) and RXRα WT-3’-UTR (50ng/well) using X-tremeGENE Transfection Reagents (Roche, USA). Mimic control and RXRα Mut-3’-UTR were used as positive and negative controls, respectively. After 24 hours, Firefly and Renilla luciferase activities were measured sequentially using the dual luciferase assay kit (Promega, Madison, USA) following the specification on Tecan Infinite F500 (Tecan Systems, CA, USA). The Firefly luciferase activities were normalized with those of Renilla luciferase and calculated as relative Rluc/Luc Ratio. The results were presented as the mean ± SD from three independent experiments with each experiment in triplicate.

### Cloning of RXRα 3’UTR and RNA binding protein immuno- precipitation (RIP) assay

A RXRα 3’UTR was amplified from CRC cell line-HCT116 cDNAs and cloned into the XhoI/NotI site of a psiCHECK-2 vector (Promega, Madison, WI). For mutagenesis of the miR-27a-3p binding site, a QuickChange site-directed Mutagenesis Kit (Agilent Technologies, Palo Alto, CA) was used according to the manufacturer’s instructions. RIP experiments were performed using a Magna RIP™ RNA-Binding Protein Immunoprecipitation Kit (Millipore) according to the manufacturer’s instructions. Briefly, cells were harvested and lysed in RIP Lysis Buffer. RNAs were immunoprecipitated with an antibody against RBP and protein A/G magnetic beads. The magnetic bead bound complexes were immobilized with a magnet and unbound materials were washed off. Then, RNAs were extracted and analyzed by quantitative real time-PCR. The specific primers for RXRα were followed: forward: 5′-CAG CTG CAT TCT CCC ATG AG-3′; reverse: 5′-GTT CAT AGG TGA GCT GAG CTG-3′. Antibody for RIP assays of AGO2 was purchased from Cell Signaling Technology (2897S).

### Co-Immunoprecipitation and immunoblotting analysis

Cells lysates were incubated with 5ug antibody on a rotator overnight at 4°C. The protein-antibody-protein A/G agarose complexes were prepared by adding protein A/G agarose beads (Invitrogen) for an hour at 4°C. After extensive washing with RIPA lysis buffer, the immnoprecipitated complexes were resuspended in reducing sample buffer and boiled for 10 minutes. After centrifugation to pellet the agarose beads, supernatants were subjected to SDS-PAGE and immunoblotting.

### Luciferase reporter assays

To examine the effect of RXRα on β-catenin/TCF/LEF signaling, cells were plated in 96-well plate at a density of 1×10^4^ cells/well and incubated overnight in FBS-supplemented medium. Cells were transiently transfected with RXRα expression plasmid using Lipofectamine 2000. After 6 hours, cells were transfected with β-catenin-TCF luciferase reporter construct-TOPFlash (Upstate Biotechnology, Lake Placid, NY). Luciferase activity was measured with the Dual Luciferase reporter assay system (Promega Corporation, Madison, WI).

### Xenograft tumor model

Female BALB/c-nude mice (4-5 weeks old and weighing 15-18g) were housed under pathogen-free conditions. SW480 and HCT116 cells were trypsinized, washed twice with serum-free medium and reconstituted in serum-free medium DMEM, mixed 1:1 with Matrigel (Becton-Dickinson) and then inoculated subcutaneously into the right flank of each nude mouse. A local miR-27a-3p antagomir or agomir treatment was initiated when the tumor was palpable at a volume of approximately 20 mm^3.^ The mice were randomly assigned into treatment and negative control groups (n=6 mice/group) and given intratumour injection with 2nM miR-27a-3p antagomir or agomir or non-specific control miRNA dissolved in 30μl PBS every 3 days. We modified antagomir or agomir with 2-O-methyl and conjugated cholesterol to the ends of the antagomir or agomir, which can retain full potency of the antagomir or agomir, confers substantial nuclease resistance, improves bio-distribution and facilitates entry into cells. The treatment time was 12 days. Tumor size was measured every 3 days, using a digital caliper, and the tumor volume was calculated according to the formula: tumor volume (mm^3^) = length×width^2^×0.5. At the end of the experiment, all mice were sacrificed and the total weights, tumor weights, and the tumor volumes were recorded. All the experiments were performed following the Guide for the Care and Use of Laboratory Animals (National Institutes of Health publication).

As for CRC metastatic animal model, after transient transfection for 24 hours, HT29 cells were trypsinized, washed twice with serum-free medium DMEM and reconstituted in serum-free medium DMEM and then introduced through tail-vein injection (10^6^ cells/mouse,6 mice/group). Subsequently, miR-27a-3p agomir or negative control (10nmol per mouse) was injected into the lateral vein in the tails of the nude BALB/c mice once every 6 days. Mice were sacrificed at 39 days (after 6 times injection) and lung, liver, and kidney tissues were fixed in 4% neutral formaldehyde and performed with haematoxylin and eosin staining. The CRC metastatic tumors were calculated by grossly and histological observation.

### Statistical analyses

Groups from cell culture and *in vivo* experiments were compared using an unpaired, two-tailed Student’s tests and results were presented as mean ± SD. For CCK8 assay, comparison was done by univariate variance analysis (two-way ANOVA). Statistical analyses were performed using SPSS 16.0 statistical software. p<0.05 was considered to be statistically significant.
